# 1,8-Bis(3-chloro­anilino)-*N*,*N*′-bis­(3-chloro­phen­yl)octane-1,8-diimine

**DOI:** 10.1107/S1600536811004612

**Published:** 2011-02-12

**Authors:** B. Thimme Gowda, Sabine Foro, Vinola Z. Rodrigues, H. S. Spandana, Hartmut Fuess

**Affiliations:** a Department of Chemistry, Mangalore University, Mangalagangotri 574 199, Mangalore, India; b Institute of Materials Science, Darmstadt University of Technology, Petersenstrasse 23, D-64287 Darmstadt, Germany

## Abstract

There are two half-mol­ecules in the asymmetric unit of the title compound, C_32_H_30_Cl_4_N_4_, in both of which the N—H bonds are *syn* to the *meta*-chloro substituents in the adjacent benzene ring. The other two Cl atoms of these two mol­ecules are disordered with occunpancy ratios of 0.79 (2):0.21 (2) and 0.68 (1):0.32 (1). Adjacent chloro­phenyl rings make dihedral angles of 74.3 (2) and 63.0 (2)° in the two mol­ecules. In the crystal, inter­molecular N—H⋯N hydrogen bonds link the mol­ecules into infinite chains.

## Related literature

For our study on the effect of substituents on the structures of this class of compounds, see: Gowda *et al.* (2007 ▶, 2009 ▶, 2010 ▶). 
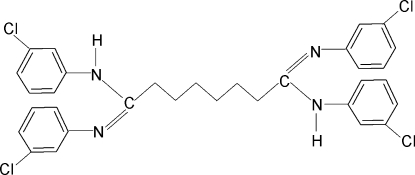


## Experimental

### 

#### Crystal data

C_32_H_30_Cl_4_N_4_
*M* *_r_* = 612.40
Monoclinic, 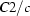

*a* = 22.349 (3) Å
*b* = 13.223 (2) Å
*c* = 22.644 (3) Å
β = 108.79 (1)°
*V* = 6335.1 (15) Å^3^
*Z* = 8
Cu *K*α radiation
μ = 3.61 mm^−1^
*T* = 299 K
0.35 × 0.28 × 0.25 mm

#### Data collection

Enraf–Nonius CAD-4 diffractometer
Absorption correction: ψ scan (North *et al.*, 1968 ▶) *T*_min_ = 0.365, *T*_max_ = 0.466
11300 measured reflections
5657 independent reflections
3976 reflections with *I* > 2σ(*I*)
*R*_int_ = 0.057
3 standard reflections every 120 min  intensity decay: 1.0%

#### Refinement

*R*[*F* ^2^ > 2σ(*F* ^2^)] = 0.080
*wR*(*F* ^2^) = 0.251
*S* = 1.13
5657 reflections
381 parameters
41 restraints
H-atom parameters constrained
Δρ_max_ = 1.08 e Å^−3^
Δρ_min_ = −0.80 e Å^−3^

### 

Data collection: *CAD-4-PC* (Enraf–Nonius, 1996 ▶); cell refinement: *CAD-4-PC*; data reduction: *REDU4* (Stoe & Cie, 1987 ▶); program(s) used to solve structure: *SHELXS97* (Sheldrick, 2008 ▶); program(s) used to refine structure: *SHELXL97* (Sheldrick, 2008 ▶); molecular graphics: *PLATON* (Spek, 2009 ▶); software used to prepare material for publication: *SHELXL97*.

## Supplementary Material

Crystal structure: contains datablocks I, global. DOI: 10.1107/S1600536811004612/bq2276sup1.cif

Structure factors: contains datablocks I. DOI: 10.1107/S1600536811004612/bq2276Isup2.hkl

Additional supplementary materials:  crystallographic information; 3D view; checkCIF report

## Figures and Tables

**Table 1:** Hydrogen-bond geometry (Å, °)

*D*—H⋯*A*	*D*—H	H⋯*A*	*D*⋯*A*	*D*—H⋯*A*
N2—H2*A*⋯N3	0.86	2.24	3.049 (4)	157
N4—H4*A*⋯N1^i^	0.86	2.23	3.072 (4)	168

Symmetry code: (i) 
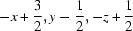
.

**Fig. 1.**
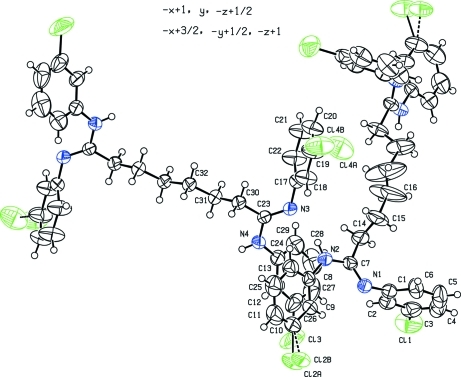
Molecular structure of (I), showing the atom labeling and displacement ellipsoids drawn at the 50% probability level. Both disorder components are shown. The minor disorder components are shown with dashed bonds. Symmetry codes for the unlabeled atoms: -x+1, y, -z+1/2 and -x+3/2, -y+1/2, -z+1.

**Fig. 2.**
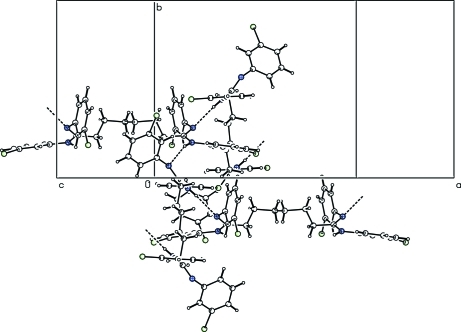
Molecular packing of (I) with hydrogen bonding shown as dashed lines. For more clarity the minor disorder components were omitted.

## supplementary crystallographic information

### Comment

The amidine moiety is an important constituent of many biologically significant compounds. As a part of studying the effect of substitutions on the structures of this class of compounds (Gowda *et al.*, 2007; 2009; 2010), the crystal structure of *N^1^*,*N^1^*-Bis(3-chlorophenyl)- *N^2^*,*N^2^*-bis(3-χhlorophenyl)-suberamidine has been determined (I) (Fig. 1). The conformations of N—H bond is *syn* to the *meta*-chloro substituent in the adjacent benzene ring.

The torsion angles of C1—N1—C7—N2, C1—N1—C7—C14, C8—N2—C7—N1 C8—N2—C7—C14, N1—C7—C14—C15 and N2—C7—C14—C15 are 172.6 (3)°, -12.9 (6)°, -8.8 (6)°, 176.2 (4)°, -86.5 (5)° and 88.4 (4)°, while those of C17—N3—C23—N4, C17—N3—C23—C30, C24—N4—C23—N3, C24—N4—C23—C30, N3—C23—C30—C31 and N4—C23—C30—C31 are 172.7 (3)°, -11.1 (5)°, -14.3 (5)°, 169.0 (3)°, 128.2 (4)° and -55.5 (4)°.

The conformations of the amine groups with respect to the attached phenyl rings are given by the torsion angles of C2—C1—N1—C7 = -80.6 (4), C6—C1—N1—C7 = 106.1 (4), C13—C8—N2—C7 = 153.8 (4), C9—C8—N2—C7 = -27.9 (5), C18—C17—N3—C23 = 103.0 (5), C22—C17—N3—C23 = -80.3 (5), C29—C24—N4—C23 = -41.3 (6), C25—C24—N4—C23 = 143.0 (4)

In the structure, two sets of phenyl rings make inter planar angles of 74.3 (2)° (C1/C6 and C8/C13 rings) and 63.0 (2)° (C17/C22 and C24/C29 rings). Further, C1/C6 phenyl ring makes a dihedral angle of 88.5 (2)° with the plane of the aliphatic group N1—C7—C14—C15—C16 and C8/C13 ring makes the angle of 20.7 (5)° with the plane of the group N2—C7—C14—C15—C16, while C17/C22 phenyl ring makes a dihedral angle of 78.9 (4)° with the plane of the group, N3—C23—C30—C31—C32 and C24/C29 ring makes the angle of 75.1 (2)° with the plane of the group, N4—C23—C30—C31—C32.

Atoms Cl2 and Cl4 in (I) are disordered and were refined using a split model. The site-occupation factors were refined so that their sum was unity [0.79 (2) and 0.22 (2) for Cl2, 0.68 (1) and 0.32 (1) for Cl4, respectively]. The corresponding bond distances in the disordered groups were restrained to be equal.

The intermolecular N–H···N hydrogen bonds (Table 1) link the molecules into infinite chains (Fig. 2).

### Experimental

Suberic acid (0.2 mol) was heated with Phosphorus oxychloride (1.2 mol) at 70°C for 2 h. The acid chloride obtained was treated with 3-chloroaniline (0.8 mol). The product obtained was added to crushed ice to obtain the precipitate. It was thoroughly washed with water and then with saturated sodium bicarbonate solution and washed again with water. It was then given a wash with 2 N HCl. It was again washed with water, filtered, dried and recrystallized from ethanol.

Prism like colorless single crystals of the title compound used in x-ray diffraction studies were obtained by a slow evaporation of its solution at room temperature.

### Refinement

The H atoms of the NH groups were located in a difference map and later restrained to the distance N—H = 0.86 (2) Å. The other H atoms were positioned with idealized geometry using a riding model with C—H = 0.93–0.97 Å. All H atoms were refined with isotropic displacement parameters (set to 1.2 times of the *U*_eq_ of the parent atom).

Atoms Cl2 and Cl4 are disordered and were refined using a split model. The corresponding site-occupation factors were refined so that their sum was unity [0.79 (2) and 0.21 (2) for Cl2, 0.68 (1) and 0.32 (1) for Cl4, respectively] and their corresponding bond distances in the disordered groups were restrained to be equal. The U^ij^ components of Cl2, Cl4, C16 and C27 were restrained to approximate isotropic behavior.

### Crystal data

**Table:**

C_32_H_30_Cl_4_N_4_	*F*(000) = 2544
*M**_r_* = 612.40	*D*_x_ = 1.284 Mg m^−^^3^
Monoclinic, *C*2/*c*	Cu *K*α radiation, λ = 1.54180 Å
Hall symbol: -C 2yc	Cell parameters from 25 reflections
*a* = 22.349 (3) Å	θ = 4.1–18.3°
*b* = 13.223 (2) Å	µ = 3.61 mm^−^^1^
*c* = 22.644 (3) Å	*T* = 299 K
β = 108.79 (1)°	Prism, colourless
*V* = 6335.1 (15) Å^3^	0.35 × 0.28 × 0.25 mm
*Z* = 8	

### Data collection

**Table:**

Enraf–Nonius CAD-4 diffractometer	3976 reflections with *I* > 2σ(*I*)
Radiation source: fine-focus sealed tube	*R*_int_ = 0.057
graphite	θ_max_ = 67.0°, θ_min_ = 3.9°
ω/2θ scans	*h* = −26→26
Absorption correction: ψ scan (North *et al.*, 1968)	*k* = −15→0
*T*_min_ = 0.365, *T*_max_ = 0.466	*l* = −27→27
11300 measured reflections	3 standard reflections every 120 min
5657 independent reflections	intensity decay: 1.0%

### Refinement

**Table:**

Refinement on *F*^2^	Primary atom site location: structure-invariant direct methods
Least-squares matrix: full	Secondary atom site location: difference Fourier map
*R*[*F*^2^ > 2σ(*F*^2^)] = 0.080	Hydrogen site location: inferred from neighbouring sites
*wR*(*F*^2^) = 0.251	H-atom parameters constrained
*S* = 1.13	*w* = 1/[σ^2^(*F*_o_^2^) + (0.1192*P*)^2^ + 8.1108*P*] where *P* = (*F*_o_^2^ + 2*F*_c_^2^)/3
5657 reflections	(Δ/σ)_max_ = 0.012
381 parameters	Δρ_max_ = 1.08 e Å^−^^3^
41 restraints	Δρ_min_ = −0.80 e Å^−^^3^

### Special details

**Table:**

Geometry. All e.s.d.'s (except the e.s.d. in the dihedral angle between two l.s. planes) are estimated using the full covariance matrix. The cell e.s.d.'s are taken into account individually in the estimation of e.s.d.'s in distances, angles and torsion angles; correlations between e.s.d.'s in cell parameters are only used when they are defined by crystal symmetry. An approximate (isotropic) treatment of cell e.s.d.'s is used for estimating e.s.d.'s involving l.s. planes.
Refinement. Refinement of *F*^2^ against ALL reflections. The weighted *R*-factor *wR* and goodness of fit *S* are based on *F*^2^, conventional *R*-factors *R* are based on *F*, with *F* set to zero for negative *F*^2^. The threshold expression of *F*^2^ > σ(*F*^2^) is used only for calculating *R*-factors(gt) *etc*. and is not relevant to the choice of reflections for refinement. *R*-factors based on *F*^2^ are statistically about twice as large as those based on *F*, and *R*- factors based on ALL data will be even larger.

### Fractional atomic coordinates and isotropic or equivalent isotropic displacement parameters (Å^2^)

**Table:**

	*x*	*y*	*z*	*U*_iso_*/*U*_eq_	Occ. (<1)
C1	0.63535 (16)	0.8110 (3)	0.10493 (18)	0.0540 (9)	
C2	0.61041 (18)	0.7419 (4)	0.0579 (2)	0.0638 (11)	
H2	0.6206	0.6737	0.0650	0.077*	
C3	0.57069 (19)	0.7723 (5)	0.0006 (2)	0.0774 (14)	
C4	0.5548 (2)	0.8728 (5)	−0.0114 (3)	0.0926 (18)	
H4	0.5283	0.8933	−0.0504	0.111*	
C5	0.5787 (2)	0.9407 (5)	0.0347 (3)	0.0955 (19)	
H5	0.5678	1.0085	0.0272	0.115*	
C6	0.6187 (2)	0.9125 (4)	0.0929 (3)	0.0751 (13)	
H6	0.6344	0.9609	0.1238	0.090*	
C7	0.66481 (15)	0.7380 (3)	0.20450 (18)	0.0492 (8)	
C8	0.77434 (16)	0.6888 (3)	0.26857 (18)	0.0478 (8)	
C9	0.80067 (18)	0.6761 (3)	0.2217 (2)	0.0620 (10)	
H9	0.7756	0.6764	0.1799	0.074*	
C10	0.8655 (2)	0.6627 (4)	0.2385 (3)	0.0802 (14)	
C11	0.9041 (2)	0.6637 (5)	0.2994 (3)	0.0901 (17)	
H11	0.9477	0.6566	0.3095	0.108*	
C12	0.8765 (2)	0.6757 (4)	0.3453 (3)	0.0830 (15)	
H12	0.9015	0.6754	0.3870	0.100*	
C13	0.8120 (2)	0.6880 (3)	0.3298 (2)	0.0657 (11)	
H13	0.7939	0.6958	0.3611	0.079*	
C14	0.59767 (18)	0.7315 (4)	0.2054 (2)	0.0710 (13)	
H14A	0.5928	0.6712	0.2278	0.085*	
H14B	0.5690	0.7264	0.1631	0.085*	
C15	0.5809 (3)	0.8240 (6)	0.2367 (3)	0.117 (2)	
H15A	0.5921	0.8852	0.2190	0.140*	
H15B	0.6038	0.8231	0.2810	0.140*	
C16	0.5112 (4)	0.8207 (10)	0.2255 (3)	0.199 (4)	
H16A	0.4926	0.8784	0.1996	0.238*	
H16B	0.4949	0.7606	0.2011	0.238*	
C17	0.63312 (17)	0.6138 (3)	0.38310 (17)	0.0518 (9)	
C18	0.6569 (2)	0.7033 (4)	0.4113 (2)	0.0776 (14)	
H18	0.6913	0.7335	0.4038	0.093*	
C19	0.6292 (3)	0.7482 (4)	0.4511 (3)	0.0897 (16)	
C20	0.5771 (2)	0.7112 (5)	0.4608 (2)	0.0844 (15)	
H20	0.5567	0.7467	0.4842	0.101*	
C21	0.5554 (3)	0.6213 (5)	0.4355 (3)	0.0998 (19)	
H21	0.5220	0.5910	0.4449	0.120*	
C22	0.5819 (3)	0.5725 (4)	0.3954 (3)	0.0885 (17)	
H22	0.5649	0.5117	0.3769	0.106*	
C23	0.69546 (16)	0.4917 (3)	0.35604 (16)	0.0447 (8)	
C24	0.68501 (19)	0.4508 (3)	0.24635 (17)	0.0528 (9)	
C25	0.7241 (2)	0.4475 (3)	0.21015 (19)	0.0644 (11)	
H25	0.7677	0.4434	0.2285	0.077*	
C26	0.6968 (4)	0.4506 (4)	0.1452 (2)	0.0906 (18)	
C27	0.6336 (4)	0.4577 (5)	0.1182 (3)	0.104 (2)	
H27	0.6162	0.4606	0.0750	0.125*	
C28	0.5958 (3)	0.4607 (5)	0.1543 (3)	0.110 (2)	
H28	0.5522	0.4660	0.1356	0.132*	
C29	0.6208 (2)	0.4560 (4)	0.2186 (2)	0.0784 (13)	
H29	0.5942	0.4564	0.2429	0.094*	
C30	0.72564 (18)	0.4535 (3)	0.42161 (16)	0.0509 (8)	
H30A	0.7085	0.4913	0.4492	0.061*	
H30B	0.7706	0.4673	0.4342	0.061*	
C31	0.71619 (18)	0.3416 (3)	0.43018 (17)	0.0526 (9)	
H31A	0.7307	0.3034	0.4008	0.063*	
H31B	0.6715	0.3281	0.4211	0.063*	
C32	0.75182 (19)	0.3065 (3)	0.49619 (17)	0.0524 (9)	
H32A	0.7958	0.3263	0.5067	0.063*	
H32B	0.7344	0.3400	0.5251	0.063*	
Cl1	0.53949 (8)	0.68487 (18)	−0.05730 (8)	0.1244 (7)	
Cl2A	0.8972 (3)	0.6372 (10)	0.1775 (3)	0.125 (2)	0.79 (2)
Cl2B	0.9069 (5)	0.695 (2)	0.1895 (8)	0.092 (6)	0.21 (2)
Cl3	0.74629 (14)	0.44327 (16)	0.10060 (9)	0.1560 (10)	
Cl4A	0.6688 (3)	0.8514 (4)	0.4962 (3)	0.123 (2)	0.677 (14)
Cl4B	0.6346 (7)	0.8849 (7)	0.4577 (9)	0.154 (4)	0.323 (14)
N1	0.68110 (12)	0.7822 (2)	0.16139 (14)	0.0510 (7)	
N2	0.70837 (13)	0.6998 (2)	0.25587 (14)	0.0508 (7)	
H2A	0.6941	0.6794	0.2848	0.061*	
N3	0.65826 (15)	0.5678 (2)	0.33991 (14)	0.0522 (7)	
N4	0.71358 (15)	0.4416 (2)	0.31194 (13)	0.0519 (7)	
H4A	0.7451	0.4009	0.3250	0.062*	

### Atomic displacement parameters (Å^2^)

**Table:**

	*U*^11^	*U*^22^	*U*^33^	*U*^12^	*U*^13^	*U*^23^
C1	0.0362 (16)	0.065 (2)	0.063 (2)	−0.0013 (16)	0.0191 (16)	0.0114 (19)
C2	0.049 (2)	0.075 (3)	0.072 (3)	−0.0037 (19)	0.026 (2)	0.006 (2)
C3	0.044 (2)	0.118 (4)	0.071 (3)	−0.013 (2)	0.020 (2)	0.001 (3)
C4	0.058 (3)	0.120 (5)	0.089 (4)	0.004 (3)	0.008 (3)	0.038 (4)
C5	0.062 (3)	0.090 (4)	0.120 (5)	0.011 (3)	0.009 (3)	0.040 (4)
C6	0.054 (2)	0.064 (3)	0.098 (3)	0.0056 (19)	0.013 (2)	0.016 (3)
C7	0.0392 (17)	0.050 (2)	0.064 (2)	0.0006 (14)	0.0234 (16)	0.0047 (17)
C8	0.0425 (17)	0.0381 (17)	0.063 (2)	0.0004 (14)	0.0169 (16)	0.0077 (16)
C9	0.050 (2)	0.072 (3)	0.069 (3)	0.0139 (18)	0.0263 (19)	0.016 (2)
C10	0.053 (2)	0.097 (4)	0.100 (4)	0.020 (2)	0.037 (2)	0.030 (3)
C11	0.047 (2)	0.103 (4)	0.115 (4)	0.011 (2)	0.018 (3)	0.027 (3)
C12	0.057 (2)	0.091 (4)	0.085 (3)	0.001 (2)	0.000 (2)	0.009 (3)
C13	0.061 (2)	0.067 (3)	0.065 (3)	0.003 (2)	0.016 (2)	0.003 (2)
C14	0.0414 (19)	0.096 (3)	0.082 (3)	−0.001 (2)	0.0286 (19)	0.024 (3)
C15	0.077 (3)	0.177 (7)	0.113 (5)	0.050 (4)	0.055 (3)	0.009 (5)
C16	0.107 (5)	0.317 (10)	0.190 (8)	0.058 (6)	0.072 (5)	−0.038 (7)
C17	0.054 (2)	0.055 (2)	0.0505 (19)	0.0127 (16)	0.0225 (16)	0.0046 (17)
C18	0.080 (3)	0.076 (3)	0.094 (3)	−0.015 (2)	0.053 (3)	−0.026 (3)
C19	0.109 (4)	0.082 (3)	0.101 (4)	−0.013 (3)	0.065 (3)	−0.032 (3)
C20	0.077 (3)	0.104 (4)	0.085 (3)	0.007 (3)	0.044 (3)	−0.025 (3)
C21	0.083 (3)	0.127 (5)	0.113 (4)	−0.023 (3)	0.064 (3)	−0.032 (4)
C22	0.093 (3)	0.089 (4)	0.107 (4)	−0.028 (3)	0.064 (3)	−0.032 (3)
C23	0.0500 (18)	0.0448 (18)	0.0433 (17)	0.0003 (15)	0.0206 (15)	0.0021 (15)
C24	0.072 (2)	0.0434 (19)	0.0433 (18)	0.0061 (17)	0.0195 (17)	−0.0022 (16)
C25	0.100 (3)	0.047 (2)	0.054 (2)	0.008 (2)	0.036 (2)	0.0037 (18)
C26	0.172 (6)	0.054 (3)	0.060 (3)	0.016 (3)	0.056 (3)	0.003 (2)
C27	0.152 (5)	0.081 (4)	0.058 (3)	0.024 (4)	0.003 (3)	−0.004 (3)
C28	0.112 (5)	0.107 (5)	0.079 (4)	0.022 (4)	−0.014 (4)	−0.022 (4)
C29	0.075 (3)	0.083 (3)	0.067 (3)	0.004 (2)	0.008 (2)	−0.010 (2)
C30	0.059 (2)	0.051 (2)	0.0449 (19)	0.0078 (16)	0.0199 (16)	0.0033 (16)
C31	0.056 (2)	0.054 (2)	0.049 (2)	0.0063 (16)	0.0190 (16)	0.0091 (16)
C32	0.062 (2)	0.054 (2)	0.0449 (18)	0.0097 (17)	0.0221 (16)	0.0059 (16)
Cl1	0.0920 (10)	0.187 (2)	0.0875 (10)	−0.0210 (11)	0.0193 (8)	−0.0408 (11)
Cl2A	0.0884 (18)	0.174 (6)	0.139 (2)	0.049 (3)	0.0729 (17)	0.027 (3)
Cl2B	0.056 (4)	0.138 (11)	0.096 (6)	0.032 (5)	0.043 (4)	0.032 (6)
Cl3	0.283 (3)	0.1340 (16)	0.1014 (12)	0.0364 (16)	0.1314 (17)	0.0228 (11)
Cl4A	0.159 (4)	0.116 (3)	0.124 (3)	−0.053 (2)	0.088 (3)	−0.066 (2)
Cl4B	0.195 (8)	0.116 (5)	0.171 (9)	0.002 (5)	0.088 (7)	−0.069 (5)
N1	0.0355 (14)	0.0593 (18)	0.0599 (18)	−0.0003 (12)	0.0177 (13)	0.0126 (15)
N2	0.0437 (15)	0.0574 (18)	0.0564 (17)	0.0006 (13)	0.0232 (13)	0.0100 (14)
N3	0.0661 (19)	0.0493 (17)	0.0488 (16)	0.0118 (14)	0.0293 (14)	0.0063 (14)
N4	0.0592 (17)	0.0569 (18)	0.0432 (15)	0.0162 (14)	0.0216 (13)	0.0029 (13)

### Geometric parameters (Å, °)

**Table:**

C1—C2	1.378 (6)	C17—C22	1.375 (6)
C1—C6	1.395 (6)	C17—N3	1.413 (5)
C1—N1	1.409 (5)	C18—C19	1.383 (6)
C2—C3	1.375 (6)	C18—H18	0.9300
C2—H2	0.9300	C19—C20	1.344 (7)
C3—C4	1.380 (8)	C19—Cl4A	1.763 (6)
C3—Cl1	1.718 (6)	C19—Cl4B	1.815 (10)
C4—C5	1.351 (9)	C20—C21	1.340 (8)
C4—H4	0.9300	C20—H20	0.9300
C5—C6	1.384 (7)	C21—C22	1.391 (7)
C5—H5	0.9300	C21—H21	0.9300
C6—H6	0.9300	C22—H22	0.9300
C7—N1	1.287 (5)	C23—N3	1.281 (5)
C7—N2	1.351 (5)	C23—N4	1.364 (4)
C7—C14	1.510 (5)	C23—C30	1.506 (5)
C8—C13	1.370 (6)	C24—C29	1.371 (6)
C8—C9	1.381 (6)	C24—C25	1.377 (6)
C8—N2	1.416 (4)	C24—N4	1.420 (5)
C9—C10	1.386 (6)	C25—C26	1.399 (7)
C9—H9	0.9300	C25—H25	0.9300
C10—C11	1.371 (8)	C26—C27	1.348 (9)
C10—Cl2B	1.712 (10)	C26—Cl3	1.725 (6)
C10—Cl2A	1.777 (7)	C27—C28	1.353 (10)
C11—C12	1.378 (8)	C27—H27	0.9300
C11—H11	0.9300	C28—C29	1.383 (8)
C12—C13	1.379 (6)	C28—H28	0.9300
C12—H12	0.9300	C29—H29	0.9300
C13—H13	0.9300	C30—C31	1.516 (5)
C14—C15	1.518 (8)	C30—H30A	0.9700
C14—H14A	0.9700	C30—H30B	0.9700
C14—H14B	0.9700	C31—C32	1.522 (5)
C15—C16	1.498 (10)	C31—H31A	0.9700
C15—H15A	0.9700	C31—H31B	0.9700
C15—H15B	0.9700	C32—C32^ii^	1.509 (8)
C16—C16^i^	1.355 (9)	C32—H32A	0.9700
C16—H16A	0.9700	C32—H32B	0.9700
C16—H16B	0.9700	N2—H2A	0.8600
C17—C18	1.368 (6)	N4—H4A	0.8600
			
C2—C1—C6	118.1 (4)	C17—C18—H18	120.4
C2—C1—N1	121.1 (4)	C19—C18—H18	120.4
C6—C1—N1	120.5 (4)	C20—C19—C18	122.9 (5)
C3—C2—C1	120.8 (5)	C20—C19—Cl4A	119.2 (4)
C3—C2—H2	119.6	C18—C19—Cl4A	117.6 (4)
C1—C2—H2	119.6	C20—C19—Cl4B	112.8 (5)
C2—C3—C4	121.0 (5)	C18—C19—Cl4B	116.7 (5)
C2—C3—Cl1	120.2 (5)	C21—C20—C19	117.8 (5)
C4—C3—Cl1	118.8 (4)	C21—C20—H20	121.1
C5—C4—C3	118.3 (5)	C19—C20—H20	121.1
C5—C4—H4	120.8	C20—C21—C22	121.3 (5)
C3—C4—H4	120.8	C20—C21—H21	119.4
C4—C5—C6	122.0 (5)	C22—C21—H21	119.4
C4—C5—H5	119.0	C17—C22—C21	120.3 (5)
C6—C5—H5	119.0	C17—C22—H22	119.8
C5—C6—C1	119.7 (5)	C21—C22—H22	119.8
C5—C6—H6	120.1	N3—C23—N4	119.4 (3)
C1—C6—H6	120.1	N3—C23—C30	126.0 (3)
N1—C7—N2	121.4 (3)	N4—C23—C30	114.5 (3)
N1—C7—C14	124.2 (3)	C29—C24—C25	120.0 (4)
N2—C7—C14	114.2 (3)	C29—C24—N4	122.3 (4)
C13—C8—C9	120.1 (3)	C25—C24—N4	117.5 (4)
C13—C8—N2	117.8 (3)	C24—C25—C26	118.6 (5)
C9—C8—N2	122.0 (3)	C24—C25—H25	120.7
C8—C9—C10	118.1 (4)	C26—C25—H25	120.7
C8—C9—H9	121.0	C27—C26—C25	121.1 (5)
C10—C9—H9	121.0	C27—C26—Cl3	120.9 (5)
C11—C10—C9	122.7 (5)	C25—C26—Cl3	117.9 (5)
C11—C10—Cl2B	110.7 (7)	C26—C27—C28	119.7 (5)
C9—C10—Cl2B	121.7 (5)	C26—C27—H27	120.2
C11—C10—Cl2A	120.3 (4)	C28—C27—H27	120.2
C9—C10—Cl2A	116.9 (5)	C27—C28—C29	121.0 (6)
C10—C11—C12	118.0 (4)	C27—C28—H28	119.5
C10—C11—H11	121.0	C29—C28—H28	119.5
C12—C11—H11	121.0	C24—C29—C28	119.5 (6)
C13—C12—C11	120.5 (5)	C24—C29—H29	120.2
C13—C12—H12	119.8	C28—C29—H29	120.2
C11—C12—H12	119.8	C23—C30—C31	114.6 (3)
C8—C13—C12	120.7 (4)	C23—C30—H30A	108.6
C8—C13—H13	119.7	C31—C30—H30A	108.6
C12—C13—H13	119.7	C23—C30—H30B	108.6
C7—C14—C15	110.9 (4)	C31—C30—H30B	108.6
C7—C14—H14A	109.5	H30A—C30—H30B	107.6
C15—C14—H14A	109.5	C30—C31—C32	111.9 (3)
C7—C14—H14B	109.5	C30—C31—H31A	109.2
C15—C14—H14B	109.5	C32—C31—H31A	109.2
H14A—C14—H14B	108.1	C30—C31—H31B	109.2
C14—C15—C16	107.3 (7)	C32—C31—H31B	109.2
C14—C15—H15A	110.3	H31A—C31—H31B	107.9
C16—C15—H15A	110.3	C32^ii^—C32—C31	112.6 (4)
C14—C15—H15B	110.3	C32^ii^—C32—H32A	109.1
C16—C15—H15B	110.3	C31—C32—H32A	109.1
H15A—C15—H15B	108.5	C32^ii^—C32—H32B	109.1
C16^i^—C16—C15	120.0 (11)	C31—C32—H32B	109.1
C16^i^—C16—H16A	107.3	H32A—C32—H32B	107.8
C15—C16—H16A	107.3	C7—N1—C1	120.8 (3)
C16^i^—C16—H16B	107.3	C7—N2—C8	128.9 (3)
C15—C16—H16B	107.3	C7—N2—H2A	115.6
H16A—C16—H16B	106.9	C8—N2—H2A	115.6
C18—C17—C22	118.2 (4)	C23—N3—C17	120.5 (3)
C18—C17—N3	120.9 (4)	C23—N4—C24	125.8 (3)
C22—C17—N3	120.8 (4)	C23—N4—H4A	117.1
C17—C18—C19	119.2 (4)	C24—N4—H4A	117.1
			
C6—C1—C2—C3	0.6 (6)	C19—C20—C21—C22	−6.4 (10)
N1—C1—C2—C3	−173.3 (3)	C18—C17—C22—C21	0.1 (8)
C1—C2—C3—C4	0.1 (6)	N3—C17—C22—C21	−176.5 (5)
C1—C2—C3—Cl1	−179.4 (3)	C20—C21—C22—C17	3.0 (10)
C2—C3—C4—C5	−0.8 (7)	C29—C24—C25—C26	0.6 (6)
Cl1—C3—C4—C5	178.8 (4)	N4—C24—C25—C26	176.4 (4)
C3—C4—C5—C6	0.7 (8)	C24—C25—C26—C27	0.7 (7)
C4—C5—C6—C1	0.0 (8)	C24—C25—C26—Cl3	−178.5 (3)
C2—C1—C6—C5	−0.6 (6)	C25—C26—C27—C28	−0.9 (9)
N1—C1—C6—C5	173.3 (4)	Cl3—C26—C27—C28	178.3 (5)
C13—C8—C9—C10	−0.1 (6)	C26—C27—C28—C29	−0.3 (10)
N2—C8—C9—C10	−178.0 (4)	C25—C24—C29—C28	−1.7 (8)
C8—C9—C10—C11	−1.3 (8)	N4—C24—C29—C28	−177.3 (5)
C8—C9—C10—Cl2B	−154.1 (14)	C27—C28—C29—C24	1.6 (10)
C8—C9—C10—Cl2A	175.5 (6)	N3—C23—C30—C31	128.1 (4)
C9—C10—C11—C12	1.9 (9)	N4—C23—C30—C31	−55.7 (4)
Cl2B—C10—C11—C12	157.4 (13)	C23—C30—C31—C32	175.8 (3)
Cl2A—C10—C11—C12	−174.8 (7)	C30—C31—C32—C32^ii^	−174.4 (4)
C10—C11—C12—C13	−1.1 (9)	N2—C7—N1—C1	172.6 (4)
C9—C8—C13—C12	0.7 (7)	C14—C7—N1—C1	−12.8 (6)
N2—C8—C13—C12	178.8 (4)	C2—C1—N1—C7	−80.4 (5)
C11—C12—C13—C8	−0.1 (8)	C6—C1—N1—C7	105.8 (5)
N1—C7—C14—C15	−87.0 (6)	N1—C7—N2—C8	−8.7 (6)
N2—C7—C14—C15	88.0 (5)	C14—C7—N2—C8	176.2 (4)
C7—C14—C15—C16	170.0 (5)	C13—C8—N2—C7	153.9 (4)
C14—C15—C16—C16^i^	122.4 (4)	C9—C8—N2—C7	−28.1 (6)
C22—C17—C18—C19	0.4 (8)	N4—C23—N3—C17	172.8 (3)
N3—C17—C18—C19	177.1 (5)	C30—C23—N3—C17	−11.2 (6)
C17—C18—C19—C20	−4.2 (9)	C18—C17—N3—C23	103.0 (5)
C17—C18—C19—Cl4A	169.8 (5)	C22—C17—N3—C23	−80.4 (6)
C17—C18—C19—Cl4B	−151.6 (8)	N3—C23—N4—C24	−14.6 (6)
C18—C19—C20—C21	7.1 (10)	C30—C23—N4—C24	169.0 (3)
Cl4A—C19—C20—C21	−166.8 (6)	C29—C24—N4—C23	−41.1 (6)
Cl4B—C19—C20—C21	155.6 (9)	C25—C24—N4—C23	143.1 (4)

Symmetry codes: (i) −*x*+1, *y*, −*z*+1/2; (ii) −*x*+3/2, −*y*+1/2, −*z*+1.

### Hydrogen-bond geometry (Å, °)

**Table:**

*D*—H···*A*	*D*—H	H···*A*	*D*···*A*	*D*—H···*A*
N2—H2A···N3	0.86	2.24	3.049 (4)	157
N4—H4A···N1^iii^	0.86	2.23	3.072 (4)	168

Symmetry codes: (iii) −*x*+3/2, *y*−1/2, −*z*+1/2.
